# ChIP-seq Defined Genome-Wide Map of TGFβ/SMAD4 Targets: Implications with Clinical Outcome of Ovarian Cancer

**DOI:** 10.1371/journal.pone.0022606

**Published:** 2011-07-25

**Authors:** Brian A. Kennedy, Daniel E. Deatherage, Fei Gu, Binhua Tang, Michael W. Y. Chan, Kenneth P. Nephew, Tim H-M. Huang, Victor X. Jin

**Affiliations:** 1 Department of Biomedical Informatics, The Ohio State University, Columbus, Ohio, United States of America; 2 Human Cancer Genetics Program, The Ohio State University, Columbus, Ohio, United States of America; 3 Department of Life Science, National Chung Cheng University, Min-Hsiung, Chia-Yi, Taiwan, Republic of China; 4 Medical Sciences, Indiana University School of Medicine, Bloomington, Indiana, United States of America; Duke-National University of Singapore Graduate Medical School, Singapore

## Abstract

Deregulation of the transforming growth factor-β (TGFβ) signaling pathway in epithelial ovarian cancer has been reported, but the precise mechanism underlying disrupted TGFβ signaling in the disease remains unclear. We performed chromatin immunoprecipitation followed by sequencing (ChIP-seq) to investigate genome-wide screening of TGFβ-induced SMAD4 binding in epithelial ovarian cancer. Following TGFβ stimulation of the A2780 epithelial ovarian cancer cell line, we identified 2,362 SMAD4 binding loci and 318 differentially expressed SMAD4 target genes. Comprehensive examination of SMAD4-bound loci, revealed four distinct binding patterns: 1) Basal; 2) Shift; 3) Stimulated Only; 4) Unstimulated Only. TGFβ stimulated SMAD4-bound loci were primarily classified as either Stimulated only (74%) or Shift (25%), indicating that TGFβ-stimulation alters SMAD4 binding patterns in epithelial ovarian cancer cells. Furthermore, based on gene regulatory network analysis, we determined that the TGFβ-induced, SMAD4-dependent regulatory network was strikingly different in ovarian cancer compared to normal cells. Importantly, the TGFβ/SMAD4 target genes identified in the A2780 epithelial ovarian cancer cell line were predictive of patient survival, based on in silico mining of publically available patient data bases. In conclusion, our data highlight the utility of next generation sequencing technology to identify genome-wide SMAD4 target genes in epithelial ovarian cancer and link aberrant TGFβ/SMAD signaling to ovarian tumorigenesis. Furthermore, the identified SMAD4 binding loci, combined with gene expression profiling and in silico data mining of patient cohorts, may provide a powerful approach to determine potential gene signatures with biological and future translational research in ovarian and other cancers.

## Introduction

The transforming growth factor-β (TGFβ) signaling pathway plays an important role in controlling proliferation, differentiation, and other cellular processes including the growth of ovarian surface epithelial cell (OSE) [1], [2]. Dysregulation of TGFβ signaling is frequently observed in epithelial ovarian cancer (EOC) and may be crucial to EOC development [3], [4]. The effects of TGFβ are mediated by three TGFβ ligands — TGFβ1, TGFβ2 and TGFβ3, acting through TGFβ type 1 and type 2 receptors [5]–[7]. TGFBR2 is the specific receptor for TGFβ ligands. The functional receptor complex regulates the activation of downstream Smad and non Smad pathways [8]. The phosphorylated type 1 receptor recruits and phosphorylates receptor-regulated Smads R-Smads). Of the five R-Smads in mammals, the TGFBR2–ALK5 complex activates SMAD2 and SMAD3, whereas the TGFBR2–ALK1 complex activates SMAD1, SMAD5 and SMAD8 [9]. Activated R-Smads form heteromeric complexes with the common partner Smad (co-Smad; SMAD4 in mammals) and translocate into the nucleus [6]. As the affinity of the activated Smad complex for the Smad-binding element is insufficient to support association with endogenous promoters of target genes, Smad complexes must associate with other DNA binding transcription factors to regulate expression [7]. Numerous studies have shown that various families of transcription factors, such as the forkhead, homeobox, zinc finger, LEF1, Ets, and basic helix–loop–helix (bHLH) families, can serve as SMAD4 partner proteins to achieve high affinity and selectivity for target promoters with the appropriate binding elements [10]–[14].

The A2780 human epithelial ovarian cancer cell line is sensitive to cis-diamminedichloroplatinum(II) (cisplatin), one of the platinum-type agents (carbolatin or cisplatin) used in the treatment of ovarian cancer. In addition to serving as a useful model for studying drug-sensitive disease, A2780 cells display partial TGFβ dysregulation, indicated by only a modest increase in SMAD4 expression and transduction of existing SMAD4 from the cytoplasm to the nucleus following TGFβ stimulation [15]. Thus, this cell line is also an appropriate model system for carrying out genome-wide mapping of SMAD4 target genes and identifying the deregulated TGFβ/SMAD4 target genes and pathways implicated in ovarian cancer patients.

Recent comparisons of ChIP-seq (chromatin immunoprecipitation-sequencing) to array-based approaches clearly demonstrated that ChIP-seq technology yielded higher resolution, greater depth and improved mapping accuracy of transcription factor binding and histone modifications on a genome-wide scale [16]–[18]. In the current study, we used ChIP-seq technology to study TGFβ/SMAD4 regulation in the platinum-sensitive A2780 ovarian cancer cell line. We profiled SMAD4 binding loci following with TGFβ stimulation. Using computational approaches, we have investigated the SMAD4 binding pattern and compared it with the SMAD4 binding pattern of both a normal immortalized ovarian surface epitheilial cell (IOSE) from our previous study [12] and human keratinocytes (HaCaT) from Koinuma et al [11]. Further, we generated TGFβ/SMAD4-regulated gene signatures and utilized an *in silico* mining approach to correlate the identified signatures with clinical outcome data from two publicly available ovarian cancer patient cohorts. Our integrative approach revealed significant associations of TGFβ/SMAD4 regulatory networks with both progression free and overall survival in ovarian cancer patients. By identifying thousands of SMAD4 binding loci as well as regulated genes, our data provide both a new resource for studying the mechanism underlying dysregulated TGFβ signaling in ovarian cancer cells as well as potential prognostic biomarkers for future ovarian cancer translational research.

## Results

### Genome-Wide SMAD4 occupancy defined by ChIP-seq technology

Our previous studies [12], [15], [19], [20] and others [2], [4], [21]–[23] have tried to establish and characterize the molecular mechanisms of dysregulated TGFβ-mediated signaling in ovarian cancer cells and acquired cisplatin-resistant ovarian cancer cells. In order to further elucidate the details of the underlying mechanisms, we used ChIP-seq technology to identify the genomic locations bound by SMAD4 in A2780 cells before and after TGFβ stimulation.

Using ChIP-seq, all samples were initially sequenced to generate a set of raw reads (each read has a length of 36 bp) from Illumina/Solexa GAII system (**Table S1**) ranging from ∼43 million to ∼51 million reads per sample. After mapping to UCSC Human HG18 assembly, a set of ∼26 million and ∼32 million mapped reads with unique genomic locations were obtained for Unstimulated A2780 and TGFβ-stimulated A2780 respectively. We then applied our peak-calling detection program, BELT, [24], [25] (See **Materials and Methods**) to identify the binding loci of SMAD4 in these two conditions. Briefly, our BELT program uses a percentile scoring method to determine the enrichment threshold value for each of the top percentiles from all binding regions, followed by identifying the number of binding loci at each percentile level. In order to determine the significance of each percentile, a set of randomly simulated reads is used as a background to estimate the false discovery rate (FDR). Our ChIP-seq data confirmed multiple SMAD4 binding loci previously identified in different tissues and cell types including Gadd45A, CTGF, JAG1, LEMD3 [14], MYC [26], EDN1, RYBP, DST, and BCAT1 [11].

#### Basal occupancy

**Figure 1:**
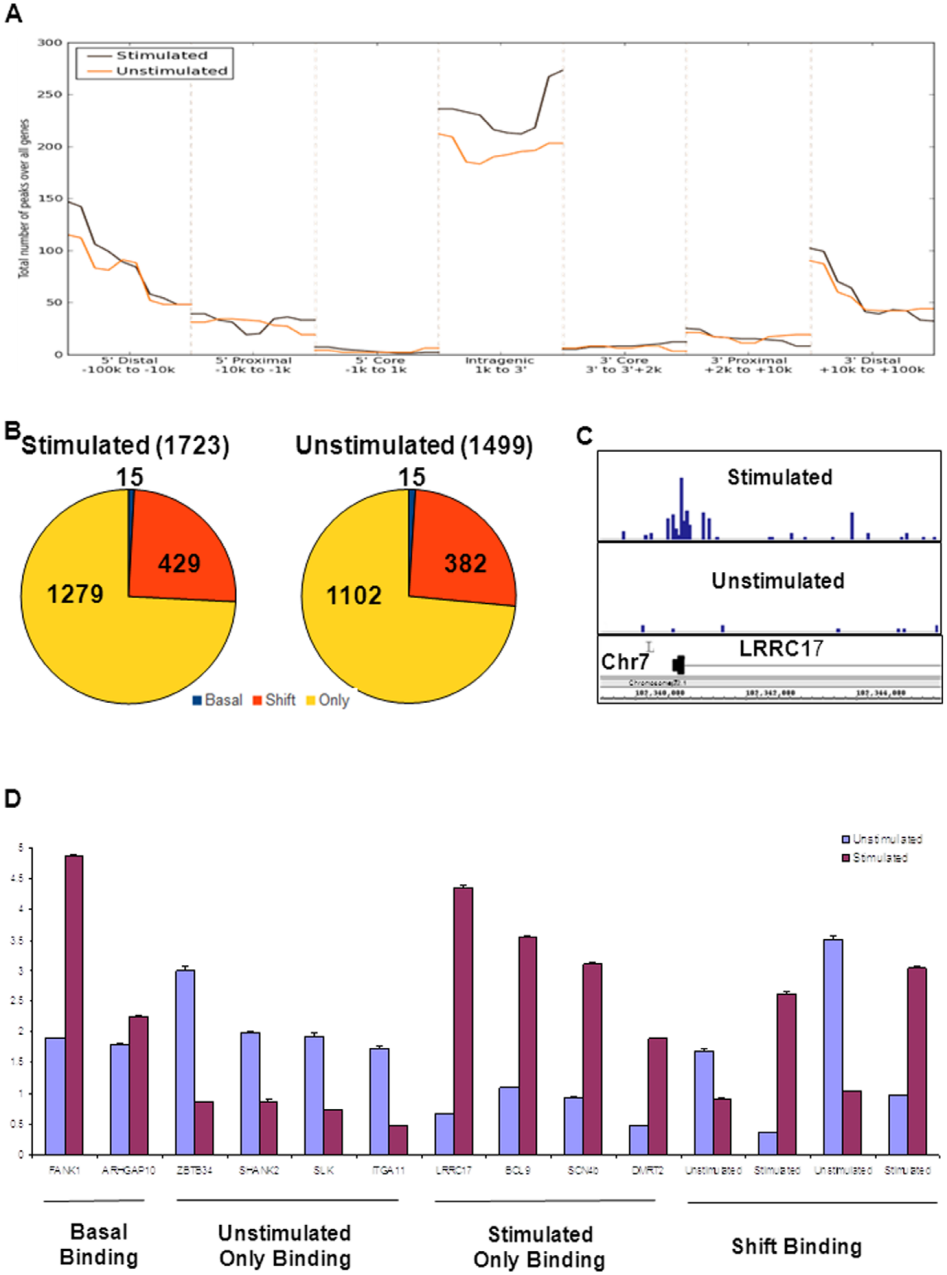
Identification of TGFβ/SMAD4 binding loci. **A**) The distribution of the location of SMAD4 binding loci in a histogram plot based on their relative to a closest known RefGene 5′TSS. **B**) Classification of SMAD4 binding loci into four binding patterns. Stimulated Only binding loci are those whose associated RefGene has binding loci only in the stimulated set, likewise for Unstimulated only. Shift binding loci have a binding loci appearing on the same gene in both conditions and they are greater than 1,000 nt apart. Basal binding loci appear on the same gene in both conditions but they are less than 1,000 nt apart. **C**) A screenshot showing LRRC17 binding pattern, where SMAD4 binds to 5′TSS of LRRC17 after TGFβ stimulation, is categorized to Stimulated Only Binding. **D**) Abundance of DNA following SMAD4 ChIP pull down as compared to DNA present following pull down with non-specific IgG antibody as determined by quantitative syber green PCR. U and S used to represent the Unstimulated and Stimulated binding regions of SLC40A1 respectively. * represents a t-test p-value of less than 0.05 and denotes significant enrichment relative to IgG control.

We identified 2,009 SMAD4 binding loci in the basal (unstimulated) condition in the A2780 cell line (**Table S1**). We found that 1,499 (74.6%) loci were located within +/−100 kb of a known RefSeq gene [27]. Surprisingly, only small portion (267 of 1499, 13.3%) were within the promoter region (+/−8 kb), of a gene while the majority of binding loci were either 10 kb upstream of the 5′TSS or 10 kb downstream 3′TSS (**Figure 1A** – red line). This unbiased whole genome wide location analysis suggested that many other previous genome-wide studies based on promoter ChIP-chip technology [11], [12], [28] may only identify subsets of SMAD4 target genes.

#### TGFβ-stimulated binding

Upon stimulation with TGFβ, 2,362 SMAD4 binding loci were identified (**Table S1**). Overall, the distribution of the location of SMAD4 binding loci after TGFβ stimulation is very similar to the one before stimulation (**Figure 1A** – black line for stimulated and red line for unstimulated). However, the binding patterns between two conditions (before and after TGFβ stimulation) are dramatically different (**Figure 1B**). We first removed these binding loci located far away from any known RefSeq genes (+/−100 kb) and then classified them (1,723 loci for stimulated and 1,499 loci for unstimulated) into four different binding patterns: 1) Basal Binding – two binding loci are associated with same gene and within 1 kb distance of each other (i.e. unchanged binding); 2) Shift Binding – two binding loci are associated with same gene in both conditions, but they are more than 1 kb apart from one another; 3) Stimulated Only Binding – a binding loci associated with a gene only in the stimulated condition; 4) Unstimulated Only Binding – a binding loci associated with a gene only in the unstimulated condition. Based on the above classification, we determined that 74.2% (1,279 of 1,723) and 73.5% (1,102 of 1,499) of the binding loci were in the Stimulated Only Binding and Unstimulated Only Binding categories respectively. While 24.8% (429 of 1,723) and 25.5 (382 of 1,499) binding loci were classified into the Shift Binding category for the stimulated and the unstimulated condition respectively, only 15 binding loci in each condition (0.9% and 1.0% respectively) fell into the Basal Binding category. Our genomic mapping results showed that TGFβ stimulation of ovarian cancer cells may alter the landscape of SMAD4 binding patterns. A complete list of classified binding patterns is shown in **Table S2**.

Further, in order to verify that TGFβ stimulation resulted in the binding changes we observed in the ChIP-seq data, we randomly chose a set of 22 targets identified by our analysis and performed ChIP-qPCR using DNA isolated from an immunoprecipitation that was distinct from the DNA used for ChIP-seq. Our ChIP-qPCR validations not only confirmed the targets identified in the ChIP-seq data but also further demonstrated that the activated exogenous TGFβ signaling is capable of producing drastic changes in SMAD4 binding patterns (**Figures 1C, 1D** and **Figures S1A,B**).

### Regulation of TGFβ-stimulated SMAD4 target gene expression in A2780

**Figure 2:**
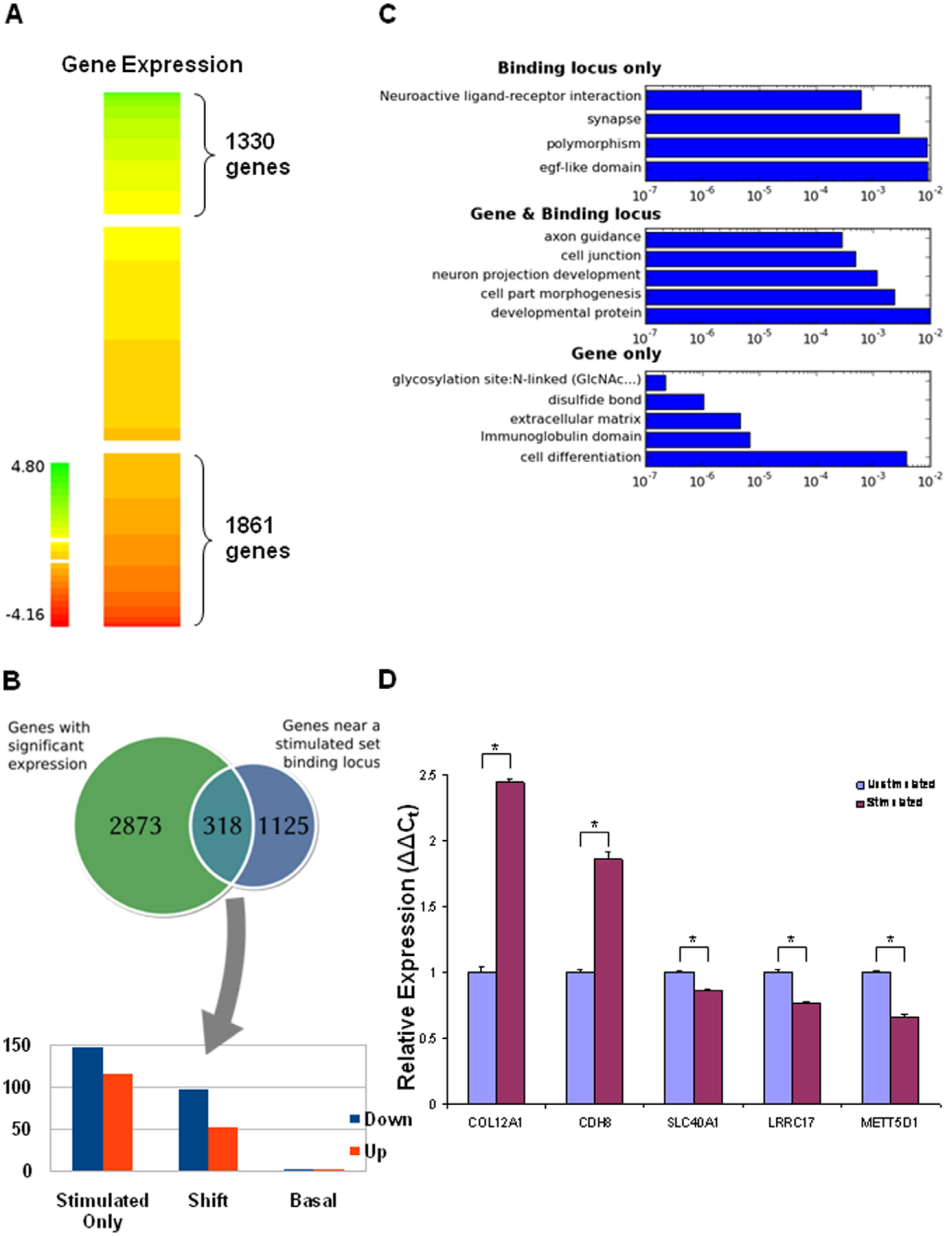
TGFβ/SMAD4 regulated genes. **A**) A heatmap of the expression fold changes for genes between the unstimulated and the TGFβ stimulated condition, showing three group of genes, up-regulated, no change, and down-regulated. Up and down regulated genes are defined as having a Log2 fold change of greater than 0.5 or less than −0.5 respectively. **B**) A comparison between the genes with SMAD4 binding loci (1443) in the TGFβ stimulated condition with all genes showing differential expression (3193), showing three different groups, those with differential expression and no SMAD4 binding loci, those with no differential expression and a SMAD4 binding loci and those with both. **C**) GO annotations for the three different group genes showing in the Venn diagram (**B**). **D**) RNA expression level as determined by qRT-PCR relative to GAPDH expression levels. Experiments were performed in biological triplicate. * represents a t-test p-value of less than 0.05 and denotes significant difference in expression between unstimulated and stimulated conditions.

Next, we performed gene expression microarrays to determine the expression status for SMAD4 target genes after TGFβ stimulation. A2780 mRNA from three independent biological replicates of both before and after 3 hours of TGFβ stimulation was prepared and assayed on Affymetrix U133 Plus 2 Platform. Overall, 3,191 genes were identified as being significantly Up or Down-regulated after TGFβ stimulation with at least a 0.5 Log2-fold change in expression and p value of less than 0.1 (**Figure 2A**). After examining the correlation with 1,443 TGFβ-stimulated SMAD4 target genes (corresponding to 1,723 SMAD4 binding loci in the stimulated condition), a majority (2,873 of 3,191) of genes with differential expression in A2780 surprisingly lacked SMAD4 binding loci, where 318 genes had at least one SMAD4 binding loci and showed at least a 0.5 Log2-fold expression change after 3 hours of TGFβ stimulation (**Figure 2B**). Details of 3,191 significantly differentially expressed genes are available in **Tables S4** and 318 genes in **Table S5**.

Gene ontology analysis showed that the differentially expressed genes with SMAD4 binding loci were significantly enriched for genes involved with cell part morphogenesis and developmental proteins (**Figure 2C**—Gene&Loci), in line with the previous studies in different cell types [12], [36]. We also found that SMAD4 binding associated genes lacking differential expression were enriched for genes with EGF-like domain and polymorphism suggesting that different signaling pathways may mediate SMAD4 functions other than TGFβ signaling (**Figure 2C**—Loci only), while the large set of differentially expressed genes lacking SMAD4 binding loci were involved in immune functions and proteinaceous extracellular matrix. After examining a more constraint p-value of 0.05 and fold change of 0.5 for TGFβ stimulated differentially expressed genes, we obtained a set of 1763 genes. Of these genes, we found 184 (10.4%) genes to have at least one TGFβ stimulated SMAD4 binding site (**Table S6**). This percentage is very similar to the dataset of which 318 (10%) of 3191 differentially expressed genes have at least a TGFβ stimulated SMAD4 binding site (**Table S5**). The GO function analysis was also very similar in top categories (**Figure S2**). To further confirm differential expressed SMAD4 targeted genes resulting from TGFβ stimulation, we randomly chose a set of 18 targets identified by our analysis and performed a RT-qPCR. More than 70% (13 of 18) genes were validated by RT-qPCR as shown in **Figure 2D** and **Figure S3**. A list of designed primers is shown in **Table S7**.

### SMAD4-dependent gene regulatory networks in TGFβ-induced ovarian cancer cells

**Figure 3:**
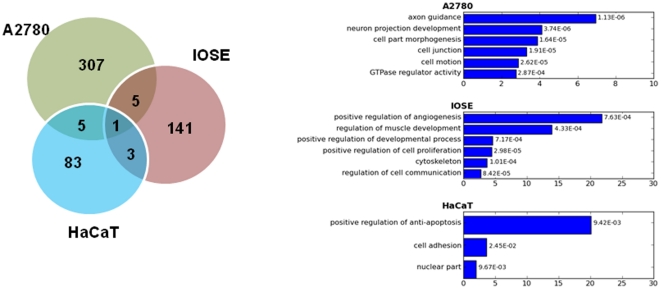
A comparison of TGFβ/SMAD4 target genes. **A**) A Venn diagram shows the comparison of TGFβ/SMAD4 target genes in three different cell types. **B**) GO annotations for the unique genes for each cell type.

Our previous study [12] and a study from Koinuma et al [11] have identified a set of 150 TGFβ stimulated SMAD4 target genes in IOSE (an immortalized ovarian surface epithelial cell line) and a set of 92 TGFβ stimulated SMAD4 target genes in HaCaT (an immortalized keratinocyte cell line). It was not surprising to find limited overlap of only 6 of 150 in IOSE, 6 of 92 in HaCaT, and 1 for all three studies in common with the 318 SMAD4 target genes in this study (**Figure 3A**) as only one, A2780, is a cancer cell line and the other two are normal cell lines. Another possibility for such low overlapping rates is that it may be due to the limited targets identified using promoter array (ChIP-promoter-chip). GO analysis [29] also showed target genes in HaCaT and IOSE were primarily involved in regulation of cell proliferation (or anti-apoptosis) and development process (muscle development), which were different from target genes in A2780 (**Figure 3B**).

**Figure 4:**
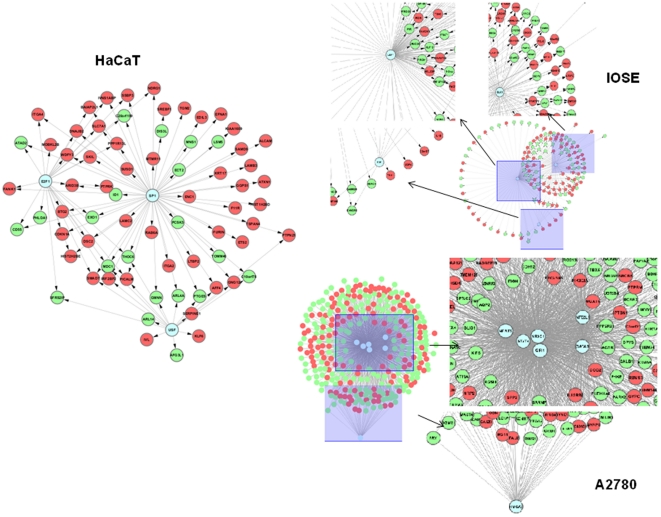
TGFβ-induced SMAD4-dependent gene regulatory networks in A) HaCaT, B) IOSE, C) A2780 cells.

To further compare the difference of the TGFβ-stimulated SMAD4-dependent gene regulatory information between these three cell types, we applied a computational analytical approach we previously developed [30] to build the SMAD4-dependent regulated networks in HaCaT, IOSE, and A2780, respectively (**Figure 4**). Briefly, our computational analytical approach started with ChIP based datasets and gene expression data. Each SMAD4 binding loci wa matched to known a RefSeq gene ID which were then be examined for differential gene expression. A set of differentially expressed SMAD4 target genes after TGFβ stimulation were further used for finding the most significant transcription factor (TF) binding partners by ChIPMotifs [31] or ChIPModudles [32], which were used as Hub TFs. The Hub TF-gene connection was determined by scanning the Hub TFs' PWMs in all binding loci and a permutation test was used to test the reliability of each connection of the network. The resulted regulatory network was visualized by Cytoscape [33].

We identified six Hub TFs, GFI1, NR3C1, SOX17, STAT4, ZNF354C, and TCF8 from 318 SMAD4-dependent target genes in A2780 cells, while four Hub TFs, LEF1 (TCF), ELK1, COUPTF (NR2F5), and E2F, were identified in IOSE cells by our previous study using a similar approach (CART model) [12]. Our computational analytical approach also identified three Hub TFs, E2F1, SP1, and USF, for 92 SMAD4-dependent target genes in HaCaT cells, which was very similar to the TF motifs identified from the Koinuma et al. study [11]. The top motif reported in their study, AP1, was missed in our results due to using an advanced classification algorithm in our ChIPModules [32] and being able to eliminate those TF motifs which are also enriched in random sets. Interestingly, we also found one Hub TF E2F (E2F1) was common between the two normal cells, but not in common with A2780 cells. Together with GO function analysis, our results indicated that E2F may act as a major SMAD4 co-transcription factor partner in mediating cell proliferation in normal cells but lost in carcinoma cells. The resultant gene regulatory networks (GRN) for all three cells are shown in **Figure 4**. Overall, our gene regulatory network analysis strongly indicates that TGFβ stimulates a different SMAD4-dependent regulatory mechanism in ovarian cancer cells compared to normal cells, i.e., the SMAD4 regulation network has become “rewired” in ovarian cancer cells.

### Gene signatures of selection and clinical outcome

**Figure 5:**
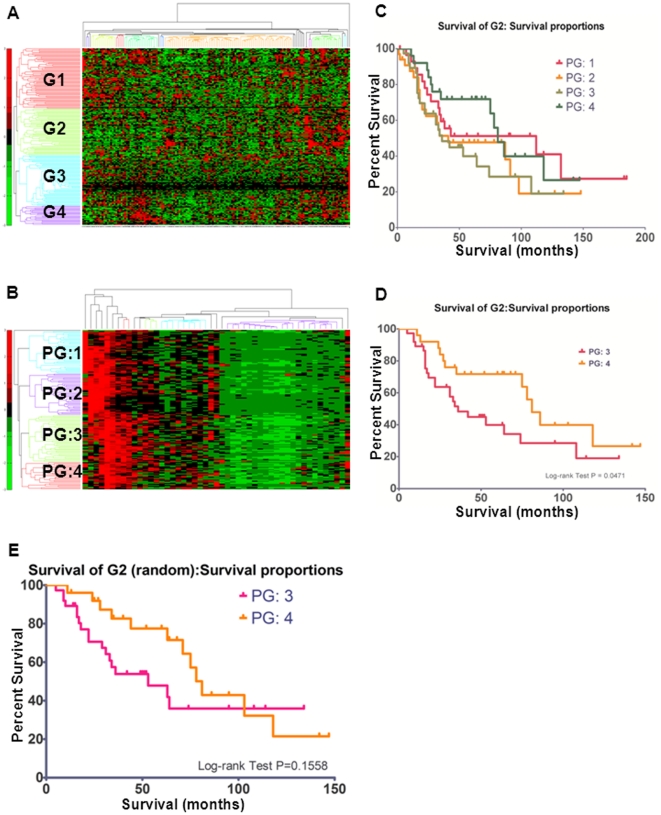
The selection of gene signatures and their associated clinical outcome. **A**) The hierarchical clustering result of the 187 genes into four gene groups, namely G1, G2, G3 and G4. The vertical axis represents the gene clusters (187 genes) and the horizontal axis stands for diverse samples (153 patients). **B**) The hierarchical clustering result of the 153 patients into four patient groups, namely PG: 1, PG: 2, PG: 3 and PG: 4 by using the G2 group of 49 genes. **C**) Survival curve plot for the G2 gene group. The horizontal axis represents the survival months and the vertical for the percent survival (%) within the corresponding patient group. Totally four patient groups, i.e. PG: 1, PG: 2, PG: 3 and PG: 4 are analyzed for the G2 gene group. **D**) A detailed survival curve plot for two patient groups, PG: 3 and PG: 4, showing a significant log-rank test p-value of 0.0471.**E**) A survival curve plot for two patient groups, PG: 3 and PG: 4 using a randomly selected 49 genes (where they are not within 49 G2 genes) showing log-rank test p-value is 0.1558.

One of the promising potential applications of genome-wide ‘omics studies using cell line systems is identification of gene signatures that can provide better prognostic information compared with standard clinical and pathological parameters [34], [35]. To address the relationship of TGFβ stimulated SMAD4-dependent target genes and clinical outcome of ovarian cancer patients, we examined the 307 target genes identified in A2780 cells in this study, which were not identified in previous studies of normal cells, in two different clinical ovarian cancer cohort studies that had reported survival data [36], [37]. We first classified the patients into different sub-groups, based on their gene signatures, and then correlated the data with the patient survival information. In mining the 153 patient cohort from Bild et al [36], we were able to use the 187 of 307 genes identified in the gene expression dataset to apply the hierarchical clustering method with distance-based measures from a trial-and-error perspective and classify the genes into four gene groups (**Figure 5A**). For each of the four gene groups, we further clustered the 153 samples into four patient groups (PGs, **Figure 5B**), and correlated the PGs with their survival information. Of 153 patients, only 124 have complete survival information, thus were further used for survival curve plots. We found that a signature of a subset of 49 genes (G2 gene group) that was able to predict a significant survival correlation for 62 of the patients with a p-value of 0.0471 (**Figure 5C,D**). Specifically PG: 4 (25 patients) displayed poor median survival of 31 months compared to PG: 3 (37 patients), with a median survival of 63 months. A survival curve plot for two patient groups, PG: 3 and PG: 4 using a randomly selected 49 genes (where they are not within 49 G2 genes) showed a log-rank test p-value of 0.1558 (**Figure 5E**). Due to limited pathological information available for this patient cohort, we were not able to significantly correlate our gene signatures with other clinical outcomes. However, a notably high percentage of stage IV patients clustered into PG3 while all stage IC and two stage IIC patients clustered into PG4, despite a similar number of stage IIIC patients in each (**Table S8**), perhaps indicating that TGFβ/SMAD4 regulated genes could be potentially used to classify a subtype of ovarian cancer patients.

When we applied the same *in silico* mining approach to the second patient cohort from Lu et al [37], (comprised of 42 patients and 5 normal people), the results showed that a gene signature of 19 of the 307 genes predicted better survival rates for PG4 and Normals than other PGs with a p-value of 0.0078 (**Figure S4**).

## Discussion

We have for the first time applied ChIP-seq technology to whole-genome-wide mapping of TGFβ-stimulated, SMAD4-dependent regulated genes in an ovarian cancer cell line (A2780). Our data show that compared to the basal state (no TGFβ stimulation), a majority of SMAD4 binding loci are either newly bound to chromatin (74.2%) or shifted bound (24.8%) upon TGFβ stimulation, suggesting TGFβ stimulated cancer cells may alter the landscape of SMAD4 binding patterns. Further, our GO analysis revealed striking similarities between the top 10 GO categories for 1,443 and 1,316 SMAD4 target genes in Stimulated and Unstimulated conditions (data not shown). However, 318 differentially expressed genes, containing at least one stimulated SMAD4 binding loci, were significantly enriched for more specific GO terms, such as cell part morphogenesis and developmental proteins. This result indicates that SMAD4 may regulate a very specific set of target genes in response to TGFβ signaling, in order to facilitate specific functions in that cell type through this specific signaling pathway. Indeed, GO analysis for SMAD4 target genes without gene expression level changes after TGFβ stimulation found one of the enriched gene categories is ‘EGF like signaling’, providing further evidence that other signaling pathways may modulate SMAD4-dependent regulated genes in ovarian cancer. One such example may be the bone morphogenetic proteins (BMPs), which are also upstream of SMAD4 and thus may be capable of regulating some of these SMAD4 target genes. BMPs have been shown to be key regulators of ovarian physiology and involved in ovarian cancer development and other cancers [38]–[40]. In future studies the contribution of each signaling pathways' regulation of the identified SMAD4 target genes will attempt to be disambiguated.

Similar to other findings for transcription factors, including estrogen receptor alpha (ERα) [41]–[43], androgen receptor (AR) [44], and peroxisome proliferator-activated receptor (PPAR) [45], we observed that a majority (>70%) of SMAD4 binding loci located more than 8 kb away from 5′TSS of a known RefSeq gene. This might suggest the TGFβ binding loci come in close proximity to the promoter through chromosome looping upon TGFβ stimulation. Interestingly, our *de novo* motif analysis also identified a SMAD-like motif in a set of 5-distal binding loci but not in a set of 5′-promoter loci (data not shown). Our genome-wide location analysis also pinpoints the importance of whole-genome-wide sequencing technologies, as we showed many binding loci are far away from the 5′TSS of a known gene and therefore a promoter-array technology may miss many target binding loci of a transcription factor. Our future studies will focus on conducting ChIP-3C-qPCR to confirm whether these distal binding loci are indeed related to these particular genes, potentially uncovering the underlying mechanism of TGFβ/SMAD4 mediated gene regulation.

One important aspect of this study is the use of *in silico* mining of publicly available patient cohort data to identify a subset of TGFβ/SMAD4 target genes as a gene signature for predicting clinical (survival) outcomes. As far as we know, this is the first study to attempt to use TGFβ signaling responsive SMAD4 regulated genes to classify ovarian cancer patients into different sub-types of patient groups, as well as predict poor survival from good survival populations with statistical significance (**Figure 5**). Thus, combining ChIP-seq identified binding loci, gene expression profiling, and an *in silico* mining of patient cohorts may provide a powerful approach for identifying potential gene signatures with biological and clinical importance.

In conclusion, our study provides the first comprehensive genome-wide map of thousands of TGFβ/SMAD4 targets in an ovarian cancer cell line, which could further be used for studying SMAD4 functions in tumorigenesis. To our knowledge, this is the first study to link TGFβ/SMAD4 regulated genes to clinical information on ovarian cancer patient survival and identify potential gene signatures for prognosis in ovarian cancer. In our future studies, we will conduct ChIP-seq analysis of TGFβ/SMAD4 binding sites using a panel of ovarian cancer cell lines representing different histological subtypes and ovarian cancer initiating cells.

## Materials and Methods

### Cell culture and TGFβ stimulation

A2780 cells [15] were cultured in RPMI 1640 (Invitrogen, Carlsbad, CA) supplemented with 10% fetal bovine serum in a 37° 5%CO_2_ incubator. Prior to TGFβ stimulation, cells were split at ∼70% confluency and inspected daily. For ChIP, 80% confluent cells were optimally stimulated with 10 ng/ml recombinant TGFβ1 (Sigma, St. Louis, MO) for 1 hour prior to formaldehyde cross-linking while expression analysis was performed after 3 hours of stimulation with 10 ng/ml TGFβ1.

### Chromatin immunoprecipitation and massive parallel sequencing

Chromatin immunoprecipitation (ChIP) was performed as previously described [46], [47] with some note worthy changes. Briefly, cells were rinsed with room temperature PBS before being cross-linked in a 1% formaldehyde solution. Cells were then harvested and homogenized in the presence of protease inhibitors before DNA was sonicated. Magnetic Dynal beads (Invitrogen) combined with a mixture of antibodies (20% SMAD4 #9515 (Cell Signaling Technology, Danvers, MA) and 80% SMAD4 DCS-46 (Santa Cruz Biotechnology, Santa Cruz, CA) were used to pull down SMAD4 overnight. Purified DNA was used to detect fold enrichment by Syber Green qRT-PCR see **Table S3** for a list of primers.

Sequencing libraries were generated for massive parallel sequencing using standard methods. Briefly, 500 ng of pulldown DNA was subjected to end repair, terminal adenylation, and adapter ligation before fragments ranging from ∼175–250 were isolated from a 2% E-gel (Invitrogen). Subsequent to a standardized 12 cycle PCR, DNA quality was evaluated on a DNA 1000 Bioanalyzer chip (Agilent Technologies, Santa Clara, CA) before being submitted for sequencing on an Illumina GAII. All ChIP-seq data is deposited in the Gene Expression Omnibus (GEO) database at National Center for Biotechnology Information (http://www.ncbi.nlm.nih.gov/geo) and are accession number GSE27526.

### Gene expression profiling

Total RNA was extracted from cells using Trizol (Invitrogen) for microrarray analysis on an Affymetrix HGU133 Plus 2 arrays (which includes more than 55,000 probes corresponding to ∼23,000 human genes on the chip) using standard hybridization and scanning protocols provided by Affymetrix (Santa Clara, CA). RNA for each sample (TGFβ-stimulated and unstimulated conditions) was isolated in biological triplicate. The RNA expression array data can be accessed through the NCBI Gene Expression Omnibus (GEO) database at National Center for Biotechnology Information through the accession number GSE27526.

### RT-qPCR and ChIP-qPCR

Total RNA was extracted from TGFβ stimulated and unstimulated cells using Trizol (Invitrogen). cDNA was then generated from 1 µg of isolated RNA through reverse transcription with a mix of oligo-dT and random hexomers in the presence of SuperScript III (Invitrogen). A Step One Plus instrument from Applied Biosystems was used in conjuncture with SYBER Green reagents (Applied Biosystems, Carlsbad, CA) to detect levels of gene expression. The ΔΔCt method of gene expression analysis was used to determine the relative level of expression as compared to the internal *GAPDH* control. A student's t-test was used to determine statistical significance of differential expression between biological triplicates. A list of specific primers used for expression analysis can be found in **Table S7**.

Our SMAD4 ChIP-seq results were confirmed via ChIP-qRTPCR. Briefly primers of approximately 150 bp were constructed to cover regions where were sequenced in the ChIP-seq experiment, and amplified with SYBER Green (Applied Biosystems). DNA was quantified on a nanodrop ND3300 (Thermo Scientific, Waltham, MA) using a Quant-iT Picogreen dsDNA Assay Kit (Invitrogen), and equal quantities of DNA were used as template. Comparison of SMAD4 and IgG pulldowns for both stimulated and unstimulated samples are reported as the average value of triplicate measurements a student's t-test was used to determine statistical significance. For a complete list of the primers used to amplify those regions see **Table S3**.

### Processing ChIP-seq and microarray gene expression data

A standard procedure for extracting image files, mapping the reads onto human genome, and filtering the mapped reads to unique reads was followed with the Solexa 1.6 pipeline. The TGFβ stimulated and unstimulated samples were each produced in two lanes of raw reads. The reads from these two lanes were combined in to a single data set. Both samples in the combined data set were processed using BELT [24], [25] developed in our laboratory with a 300 nt bin size at an acceptance threshold of 0.996 *v.s.* an input sample. (**Table S2**).

The microarray expression data was normalized using the standard protocol for the MAS5 algorithm implemented by Affymetrix in R, and a student's t-test was performed to determine the significance of the difference between the sets of biological triplicates for the stimulated and untreated samples. Significance was liberally defined as p<0.10, and a differential fold chance was defined as Log2-fold change >0.50.

### Gene regulatory network analysis

We apply our computational analytical approach developed in our laboratory [30], which includes a *de novo* method ChIPModule [32] to identify the Hub TFs for 318 TGFβ/SMAD4 genes in A2780 cells and 92 TGFβ/SMAD4 genes in HaCaT cells respectively. The Hub TFs for 150 TGFβ/SMAD4 genes in IOSE cells were from our previous study [12] which used a similar machine learning approach, CART model [48]. The gene regulatory networks were constructed by scanning the binding loci of each gene using the position weight matrix (PWM) of Hub TFs. The topology and visualization of the resulted hierarchal network is built by Cytoscape [33], where blue nodes represent Hub TFs, while red and green nodes correspond to Up and Down regulated genes respectively. The significance of the network is statistically tested by a permutation test to determine the probability of each edge of the network under random circumstances.

### Patient cohorts

The patient cohorts including gene expression and survival information for patients used in this work were from two previous studies, Bild et al [35] and Lu et al [36], which are publicly available. All gene expression data were previously normalized and were directly used in our study (153 patients in Bild et al from GSE3149 and 42 patients for Lu et al provided in their Supporting Tables).

For all hierarchical cluster analyses, log expression values of each gene were mean centered, and genes and tumors were clustered by using Pearson correlation and average linkage (MatLab). The Kaplan–Meier estimate was used to compute survival curves using logrank test, and the *p*-value of the likelihood-ratio test was used to assess statistical significance. The survival curves were generated using Prism 5 (GraphPad Software).

## Supporting Information

Figure S1 **Validation of SMAD4 binding loci.** **A**. Screenshots showing binding peaks for COL12A1 and SHANK2. **B**. Using biologically independent ChIP samples to perform ChIP-qPCR, we confirmed 22 total binding loci (patterns) for SMAD4 target genes.
(TIF)

Figure S2 **GO for 184 TGFβ/SMAD4 differentially expressed genes with a p-value of less than 0.05 showing a similar functional categories with 318 genes.** Together with RT-qPCR validations, our results demonstrated that the identified genes (318) in the study are valid for the further downstream analysis.
(TIF)

Figure S3 **Using a second ChIP sample (biologically independent) to perform RT-qPCR, we confirmed eight more differential expressed genes for TGFb/SMAD4 target genes.**
(TIF)

Figure S4 **Using TGFβ/SMAD4 regulated genes for a second cohort to predict patient survival.** **A**. A hierarchal clustering to classify 42 patients and 5 normal samples from Lu et al study using 307 SMAD4 target genes. **B**. A group of 19 gene signatures is able to predict the good survival (Normal and PG1) from bad survival.
(TIF)

Table S1 **A summary of binding sites of SMAD4 in unstimulated and TGFβ stimulated A2780 cells identified by ChIP-seq.**
(DOC)

Table S2 (XLS)

Table S3 **A list of primers designed for ChIP-qPCR.**
(DOC)

Table S4 (XLS)

Table S5 (XLS)

Table S6 (XLS)

Table S7 **A list of primers designed for RT-qPCR.**
(DOC)

Table S8 **A summary of 124 patients' tumor stages and median survival months in each groups classified by a subset of 49 TGFβ/SMAD4 gene signatures.**
(DOC)
