# Supplementary figures and images for: ChIP-seq Defined Genome-Wide Map of TGFβ/SMAD4 Targets: Implications with Clinical Outcome of Ovarian Cancer
Source: PLoS One. 2011 Jul 25;6(7):e22606. doi: 10.1371/journal.pone.0022606 (PMC3143154; doi:10.1371/journal.pone.0022606)

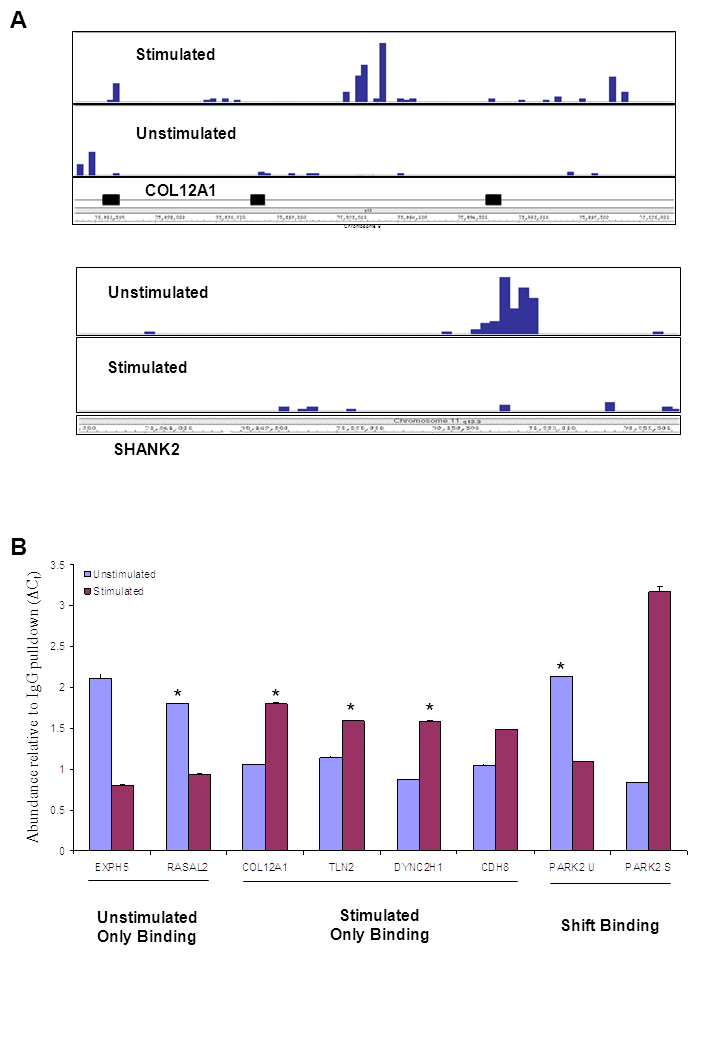

Supplement: Figure S1 — Validation of SMAD4 binding loci. A. Screenshots showing binding peaks for COL12A1 and SHANK2. B. Using biologically independent ChIP samples to perform ChIP-qPCR, we confirmed 22 total binding loci (patterns) for SMAD4 target genes. (TIF) [file pone.0022606.s001.tif]

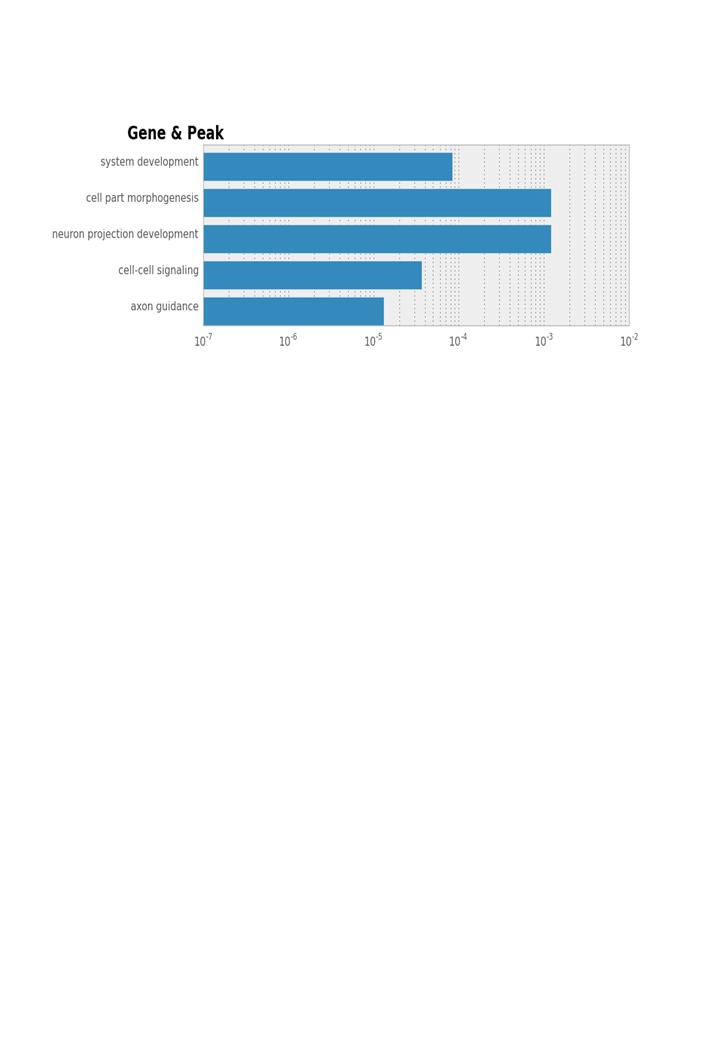

Supplement: Figure S2 — GO for 184 TGFβ/SMAD4 differentially expressed genes with a p-value of less than 0.05 showing a similar functional categories with 318 genes. Together with RT-qPCR validations, our results demonstrated that the identified genes (318) in the study are valid for the further downstream analysis. (TIF) [file pone.0022606.s002.tif]

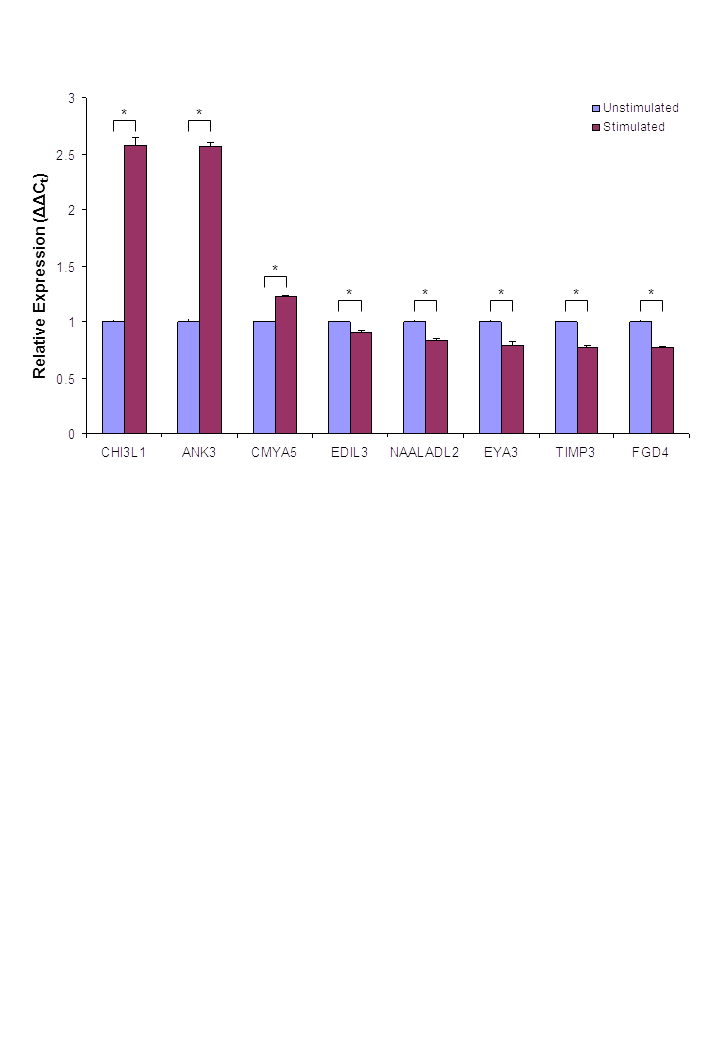

Supplement: Figure S3 — Using a second ChIP sample (biologically independent) to perform RT-qPCR, we confirmed eight more differential expressed genes for TGFb/SMAD4 target genes. (TIF) [file pone.0022606.s003.tif]

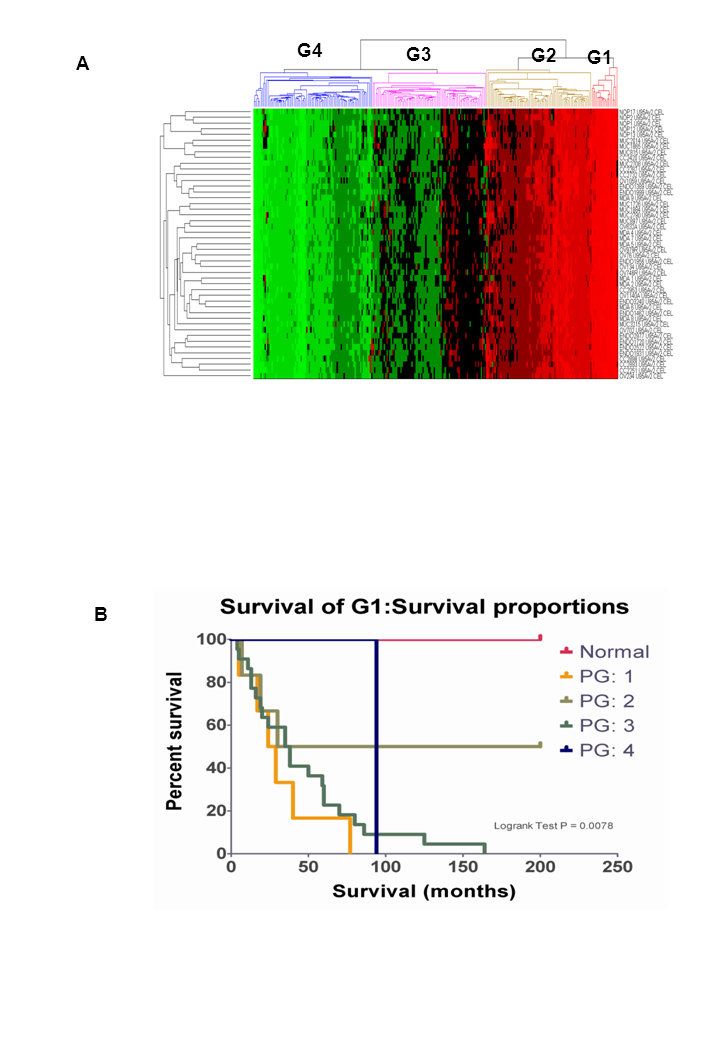

Supplement: Figure S4 — Using TGFβ/SMAD4 regulated genes for a second cohort to predict patient survival. A. A hierarchal clustering to classify 42 patients and 5 normal samples from Lu et al study using 307 SMAD4 target genes. B. A group of 19 gene signatures is able to predict the good survival (Normal and PG1) from bad survival. (TIF) [file pone.0022606.s004.tif]
