# Supplementary material for: ChIP-seq Defined Genome-Wide Map of TGFβ/SMAD4 Targets: Implications with Clinical Outcome of Ovarian Cancer
Source: PLoS One. 2011 Jul 25;6(7):e22606. doi: 10.1371/journal.pone.0022606 (PMC3143154; doi:10.1371/journal.pone.0022606)
Supplement: Table S1 — A summary of binding sites of SMAD4 in unstimulated and TGFβ stimulated A2780 cells identified by ChIP-seq. (DOC) [file pone.0022606.s005.doc]

**Table S1.** A summary of binding sites of SMAD4 in unstimulated and TGFβ stimulated A2780 cells identified by ChIP-seq.

| **Cell conditions** | **Raw Reads** | **Uniquely Mapped Reads** | **Multiple Mapped Reads** | **No Matched Reads** | **QC** | **Binding Loci^1^** |
| --- | --- | --- | --- | --- | --- | --- |
| Unstimulated A2780 | 43,113,776 | 25,745,174 | 8,472,615 | 8,160,331 | 735,656 | 2,009 |
| Stimulated A2780 | 51,600,614 | 32,278,421 | 10,554,207 | 7,823,838 | 944,148 | 2,362 |
| Input | 13,465,739 | 8,738,904 | 3,098,226 | 1,312,342 | 316,267 | - |

^1^Binding loci identified by Whole-Genome-Wide Peak Calling program (**BELT**) developed by our laboratory (see **METHODS section**) at the 99.95th percentile.
