# Supplementary material for: ChIP-seq Defined Genome-Wide Map of TGFβ/SMAD4 Targets: Implications with Clinical Outcome of Ovarian Cancer
Source: PLoS One. 2011 Jul 25;6(7):e22606. doi: 10.1371/journal.pone.0022606 (PMC3143154; doi:10.1371/journal.pone.0022606)
Supplement: Table S3 — A list of primers designed for ChIP-qPCR. (DOC) [file pone.0022606.s007.doc]

**Table S3.** A list of primers designed for ChIP-qPCR.

| **Gene Associated with binding loci** | **Forward** | **Reverse** |
| --- | --- | --- |
| BCL9 | ACAGGCATGAGCCACCACGC | CCCAGCACTTTGGGAGGCCG |
| SCN4B | TGGGGCTGGGTGGATTGGGG | CCACACAAGCCCGTGGCAGA |
| TPTE U | AGGCAGGTATCAGGCCCCGG | TGCACCTGGGGTCTGGGCTT |
| TPTE S | GCACCTGTAATGCTAATGCACTGCC | TGGCAGCAAGGGGATTGCGG |
| SLK | CCCACTCACTGCAGGCCCAC | GCAGTGAGCGCGGAAACGTG |
| ITGA11 | CACAGGTGAGGCCAGGCTGG | CCGGGTTGTCCCAGCAGCAC |
| ZBTB34 | TCCCAGGTAAGTTGGGCGTGA | TCCAGGCTGGGGTTTGTTGAGA |
| TLN2 | GGCTGCTGCTCTCAGGGGGA | TGACACAAGTCCCTCTCCGGCT |
| DYNC2H1 | AATGCTACCCCTATCACCAGTGGT | ACCTTGATGAGTGTCCTTCCACAGT |
| RASAL2 | AGGGAGGAGCTGGCGAACCA | TGCCCCACCTCTAAGCAGCT |
| FANK1 | CGGCTTAGTTGTTCTCCCGGCC | ACAGGAAATGACCAGGTGCTTCCG |
| SHANK2 | TGACTCTGGGCAAGCCACTTGATAA | TGCTCCGTGCCAGAGGCAAGA |
| LRRC17 | AACTTCCTCACACACCGCAGCA | TTGGCTTATTGCAGACGGTTCACAG |
| SLC40A1 U | GCTGCAGTGCGTCCCAAACACA | AGGGTGAACTGTCTGAGCGCCAG |
| SLC40A1 S | AACCATGTGGGAAGCTGCAGGA | TGCTCCCAGCTCAGATAGTGACC |
| ARHGAP10 | CTCCTGGATTGCCTGGCACTGC | CAGGGTGCAGACCACGTCTTGG |
| EXPH5 | TGCGTTTGGTCACACTGGTGGA | ACTTCAGGTGCTTCCCAGAAGCTCT |
| CDH8 | CTGGTGTGTTCACAGGCACATGC | ACAGCAGAGTCCCATCCCTCTCG |
| COL12A1 | AGTCCTGCTTTTCCCTTAGGAGCT | GGTGTCTTTCTGCAGTCACTTGCCA |
| DMRT2 | TGGGAGCTCAGCACACTTGGCT | GCTTGGGCACTCAGGGGATGA |
| PARK2 U | AAAGAAAGGGGCTGGTGATGCGG | ACCAAGTGTGCGCGGTAACA |
| PARK2 S | TCCACTCAGACTCACAGGGAGGGA | AGGAGGGTTGCTGAGCCTGACT |
