# Supplementary material for: ChIP-seq Defined Genome-Wide Map of TGFβ/SMAD4 Targets: Implications with Clinical Outcome of Ovarian Cancer
Source: PLoS One. 2011 Jul 25;6(7):e22606. doi: 10.1371/journal.pone.0022606 (PMC3143154; doi:10.1371/journal.pone.0022606)
Supplement: Table S7 — A list of primers designed for RT-qPCR. (DOC) [file pone.0022606.s011.doc]

**Table S7.** A list of primers designed for RT-qPCR.

| **Gene** | **Forward** | **Reverse** |
| --- | --- | --- |
| LRRC17 | GCATTGAATTCATCGATCCTGCCGC | TCCAAGGGTTATCTCTGAGCCACA |
| CDH8 | TTGGCCGGCTACACACAGACC | CCCAGTCCACTGCTTGAGCTGT |
| SLC40A1 | GATGCTGTGGATCCTTGGCCGAC | CTGCCACCACCAGCCCGTAGA |
| CHI3L1 | GCAACACTGACTATGCTGTGGGGT | GCCTCCTTGGTGAACCGGCC |
| ANK3 | TCAGCGGTGGTGAAGAGAGTGGA | GCATCTTCACTCTGCGGTAAAGCCA |
| COL12A1 | AGGCAGAAGGGATGGAGTGTCTC | CGGCCGATGCTCCATGATCCA |
| CMYA5 | GCTGCTCTCATCTCCACCAGAGG | ACGGGATTTCCGTCAGCCGT |
| EDIL3 | ACTGTCGGGTTGTTCTGAGCCTC | TTGCCTTGCTTGTCCAGCCGA |
| NAALADL2 | GGGCTTGACATCTCCAGACCGGT | TCGGTCTGGTCTCCACCCTCTCT |
| EYA3 | CCTCCTTTTGGTGCATTGTGGCC | GGGCTTGGCTGGCTTGTGGT |
| TIMP3 | TAAAGGAGGGGCCCTTCGGCA | AGACGCGACCTGTCAGCAGGTA |
| FGD4 | ACCAGCACCATGAGATGAAGGAGAC | CTGCTGGAAACGAGCCTCGGT |
| METT5D1 | CAGGCACGCAGCATCTACCCC | GAGGAAATGCTCCTGCAACGATGC |
