# Supplementary material for: ChIP-seq Defined Genome-Wide Map of TGFβ/SMAD4 Targets: Implications with Clinical Outcome of Ovarian Cancer
Source: PLoS One. 2011 Jul 25;6(7):e22606. doi: 10.1371/journal.pone.0022606 (PMC3143154; doi:10.1371/journal.pone.0022606)
Supplement: Table S8 — A summary of 124 patients' tumor stages and median survival months in each groups classified by a subset of 49 TGFβ/SMAD4 gene signatures. (DOC) [file pone.0022606.s012.doc]

**Table S8. A summary of 124 patients’ tumor stages and median survival months in each groups classified by a subset of 49 TGFβ /SMAD4 gene signatures.**

| **Patient Group** | **Median Survival months** | **Tumor Stage^1^** | | | | | | |
| --- | --- | --- | --- | --- | --- | --- | --- | --- |
|  |  | **IC (3)** | **IIC (4)** | **IIIA (3)** | **IIIB (5)** | **IIIC (91)** | **IV (17)** | **Unstage (1)** |
| **PG1 (29)** | **37** | **0** | **1** | **0** | **0** | **24** | **4** | **0** |
| **PG2 (33)** | **23** | **0** | **1** | **0** | **1** | **27** | **4** | **0** |
| **PG3 (37)** | **31** | **0** | **0** | **3** | **2** | **24** | **7** | **1** |
| **PG4 (25)** | **63** | **3** | **2** | **0** | **2** | **16** | **2** | **0** |

^1^the number in the bracket represents the number of patients in that tumor stage. 124 of 153 patients were classified by 49 gene signatures and have survival information.
